# Three-dimensional culture method enhances the therapeutic efficacies of tonsil-derived mesenchymal stem cells in murine chronic colitis model

**DOI:** 10.1038/s41598-021-98711-4

**Published:** 2021-10-01

**Authors:** Eun Mi Song, Yang Hee Joo, A. Reum Choe, Yehyun Park, Chung Hyun Tae, Ji Teak Hong, Chang Mo Moon, Seong-Eun Kim, Hye-Kyung Jung, Ki-Nam Shim, Kyung-Ah Cho, Inho Jo, Sung-Ae Jung

**Affiliations:** 1 Department of Internal Medicine, College of Medicine, Ewha Womans University, Seoul, South Korea; 2 Department of Microbiology, College of Medicine, Ewha Womans University, Seoul, South Korea; 3 Department of Molecular Medicine, College of Medicine, Ewha Womans University, Seoul, South Korea

**Keywords:** Stem cells, Gastroenterology

## Abstract

Tonsil-derived mesenchymal stem cells (TMSCs) showed therapeutic effects on acute and chronic murine colitis models, owing to their immunomodulatory properties; therefore, we evaluated enhanced therapeutic effects of TMSCs on a murine colitis model using three-dimensional (3D) culture method. The expression of angiogenic factors, VEGF, and anti-inflammatory cytokines, IL-10, TSG-6, TGF-β, and IDO-1, was significantly higher in the 3D-TMSC-treated group than in the 2D-TMSC-treated group (P < 0.05). At days 18 and 30 after inducing chronic colitis, disease activity index scores were estimated to be significantly lower in the 3D-TMSC-treated group than in the colitis control (P < 0.001 and P < 0.001, respectively) and 2D-TMSC-treated groups (P = 0.022 and P = 0.004, respectively). Body weight loss was significantly lower in the 3D-TMSC-treated group than in the colitis control (P < 0.001) and 2D-TMSC-treated groups (P = 0.005). Colon length shortening was significantly recovered in the 3D-TMSC-treated group compared to that in the 2D-TMSC-treated group (P = 0.001). Histological scoring index was significantly lower in the 3D-TMSC-treated group than in the 2D-TMSC-treated group (P = 0.002). These results indicate that 3D-cultured TMSCs showed considerably higher therapeutic effects in a chronic murine colitis model than those of 2D-cultured TMSCs via increased anti-inflammatory cytokine expression.

## Introduction

Inflammatory bowel disease (IBD) is a chronic relapsing disease that includes Crohn’s disease (CD) and ulcerative colitis (UC). The incidence and prevalence of IBD in Asian countries have increased owing to westernization of societies^1,2^. In Korea, the incidence and prevalence of IBD have continued to increase over the past three decades. In a recently published population-based cohort study, the incidence rates of CD and UC per 100,000 inhabitants were reported to increase from 0.06 and 0.29, in 1986–1990, to 2.44 and 5.82, in 2011–2015, respectively^3^.

Although the pathogenesis of IBD remains unclear, the development of IBD is thought to be associated with complex mechanisms that involve innate genetic susceptibility, aberrant immune response, gut microbiota, and other environmental factors, including westernized diet and smoking^4–6^. Over the past decades, immunosuppressive and newly developed biological agents, including anti-tumor necrosis factor α (TNF-α) agents and small molecules such as anti-integrin agent, have been employed for the treatment of IBD, in addition to conventional therapies that involve 5-aminosalicylic acid and corticosteroids, thereby improving the treatment outcomes of IBD. However, despite these improvements in IBD treatment, up to 40% of patients with IBD failed to show ideal therapeutic results^7,8^.

Recently, mesenchymal stem cells (MSCs) therapy has been introduced as a potential therapeutic alternative for several diseases, including IBD, owing to their immunosuppressive and tissue-regenerative properties^9,10^. Tonsil-derived MSCs (TMSCs) have been proposed as a new source of MSCs owing to their rapid proliferation rate and easy availability^11–13^. In our previous studies, intraperitoneal (IP) administration of TMSCs in dextran sulfate sodium (DSS)-induced acute and chronic murine colitis models demonstrated therapeutic efficacy in terms of lowering disease activity index (DAI) scores, colon length recovery, and by decreasing the expression of pro-inflammatory cytokines^14,15^. Additionally, TMSC-conditioned medium showed similar efficacy as TMSCs in acute and chronic murine colitis models, thereby suggesting that TMSCs exhibit therapeutic effects through the anti-inflammatory mechanism.

To improve the therapeutic effect of MSCs, several strategies, including pretreatment with pro-inflammatory cytokines or hypoxic condition, have been evaluated^16^. The effect of these pretreatments is thought to stimulate MSCs to maintain anti-inflammatory properties in inflamed tissues under in vivo stress conditions. Recently, a three-dimensional (3D) culture method was reported to improve survival and enhance the therapeutic properties of MSCs^17^. In an acute kidney injury model, 3D-cultured human adipose tissue-derived MSCs (ADSCs) showed increased secretion of anti-apoptotic and anti-oxidative cytokines when compared with 2D-cultured MSCs, thereby demonstrating enhanced survival and therapeutic effects. However, the therapeutic effects of 3D-cultured TMSCs in a colitis model remain unclear.

In this study, we aimed to evaluate whether 3D-culture method could improve the therapeutic efficacy of TMSCs in a chronic murine colitis model.

## Results

### In vitro

#### Increased expression of anti-inflammatory cytokines and growth factors in 3D-cultured TMSCs

**Figure 1:**
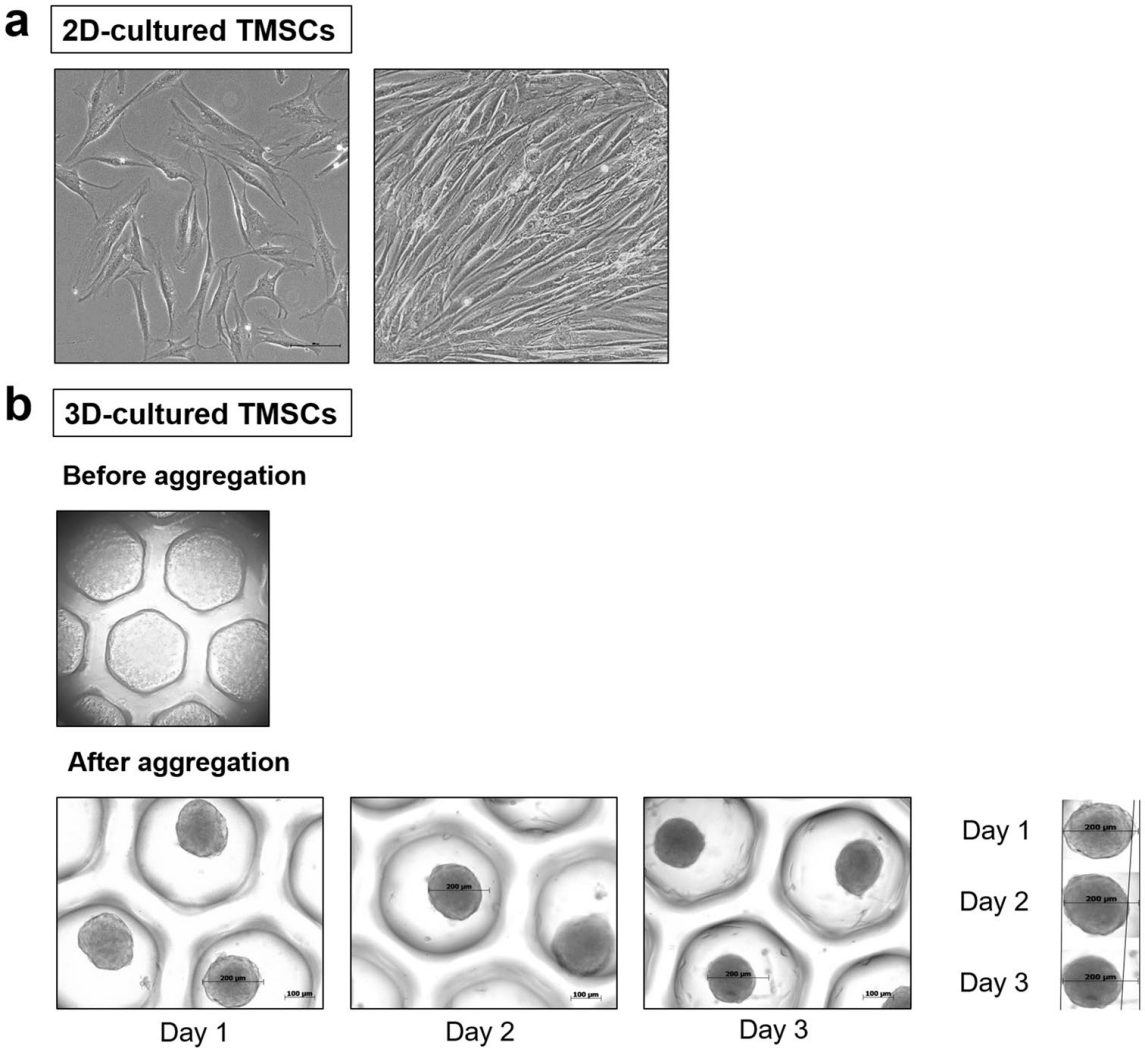
Macroscopic morphology of 2D-cultured and 3D-cultured TMSCs. (**a**) Macroscopic morphology of 2D-cultured TMSCs. (**b**) Macroscopic morphology of 3D-cultured TMSCs. 3D-cultured MSC spheroids had an average size of 182.4 µm on day 1, 169.4 µm on day 2, and 154.1 µm on day 3, and the size of spheroids gradually decreased after 2 days of culture. 2D, two-dimensional; TMSCs, tonsil-derived mesenchymal stem cells.

**Figure 2:**
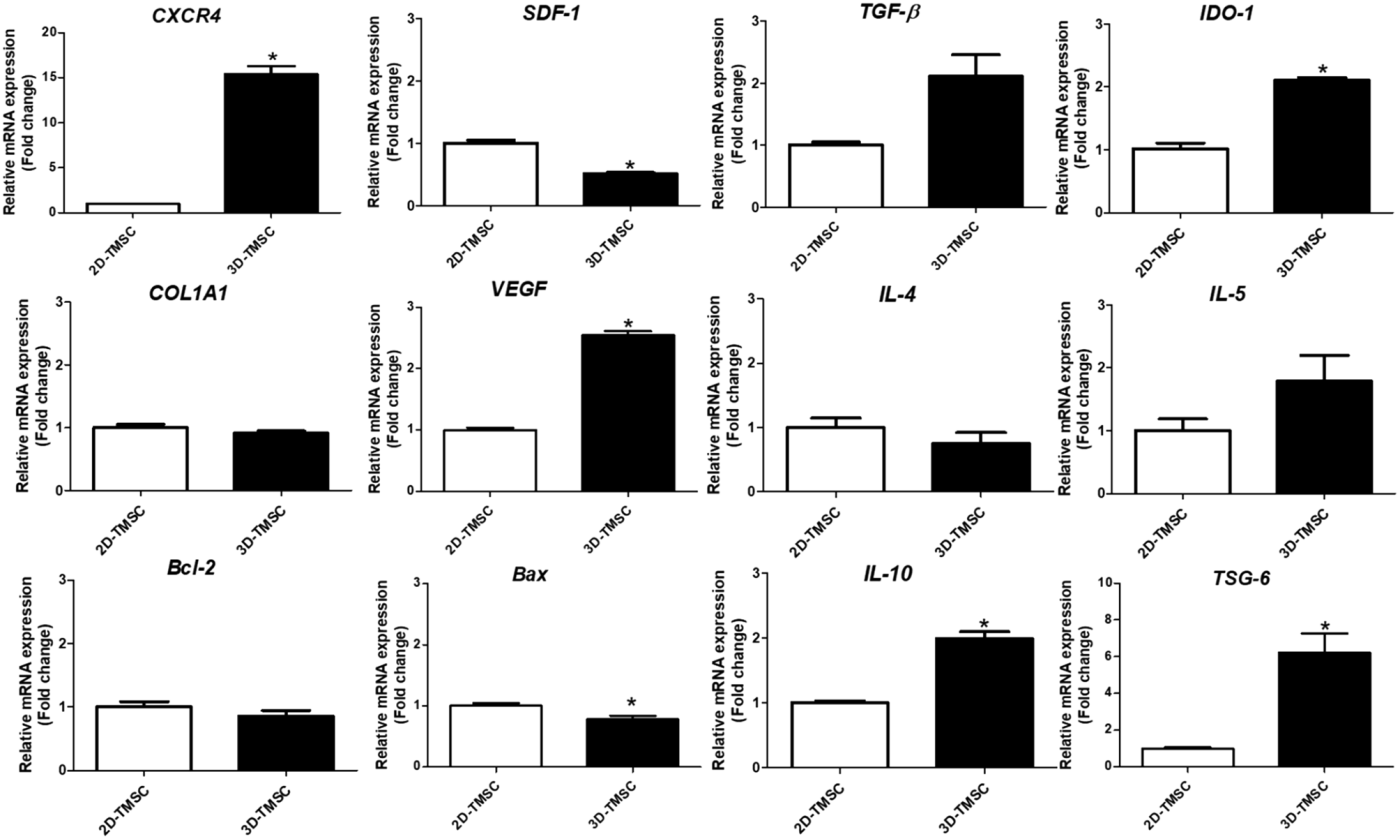
Expression of cytokines in 3D-cultured TMSCs. The expression of anti-inflammatory cytokines, including *IL-10 and TSG-6,* were significantly higher in 3D-cultured TMSCs than in 2D-cultured TMSCs as analyzed using qRT-PCR (*P < 0.005). 3D, three-dimensional; TMSCs, tonsil-derived mesenchymal stem cells; IL-10, interleukin 10; TSG-6, tumor necrosis factor-stimulated gene 6; qRT-PCR, quantitative reverse transcription quantitative polymerase chain reaction; CXCR4, C-X-C motif chemokine receptor 4; SDF-1, stromal cell-derived factor 1; TGF-β, transforming growth factor β; IDO-1, indoleamine 2,3-dioxygenase 1; VEGF, vascular endothelial growth factor; BCL-2, B-cell CLL/lymphoma 2; BAX, BCL2 associated X.

The average size of in vitro 3D-cultured TMSC spheroids on days 1, 2, and 3 was 182.4 ± 8.3, 169.4 ± 7.7, and 154.1 ± 4.9 μm, respectively (Fig. 1). The size of the spheroids significantly decreased with time after 24 h of 3D culture (Supplementary Figure 1). To evaluate the molecular characteristics of 3D-cultured TMSCs, we analyzed the expression of inflammatory and anti-inflammatory cytokines using qRT-PCR and compared them with the results obtained for 2D-cultured TMSCs. The expression of anti-inflammatory cytokines, including *interleukin* (*IL)-10* and *TNF-stimulated gene 6 (TSG-6),* was significantly higher in 3D-cultured TMSCs than in 2D-cultured TMSCs (P < 0.005, Fig. 2). Additionally, the expression level of *vascular endothelial growth factor (VEGF)* in the 3D-cultured TMSCs was significantly higher than that in the 2D-cultured TMSCs (P < 0.001). The expression levels of *C-X-C motif chemokine receptor 4 (CXCR4)* and *indoleamine 2,3-dioxygenase-1 (IDO-1)* in the 3D-cultured TMSCs were significantly higher than those in the 2D-cultured TMSCs (P < 0.005). In contrast, the expression level of *stromal cell-derived factor 1 (SDF-1)* in the 3D-cultured TMSCs was significantly lower than that in the 2D-cultured TMSCs (P < 0.005; Fig. 2).

### In vivo

#### Therapeutic effects of 3D-cultured TMSCs on chronic murine colitis models

**Figure 3:**
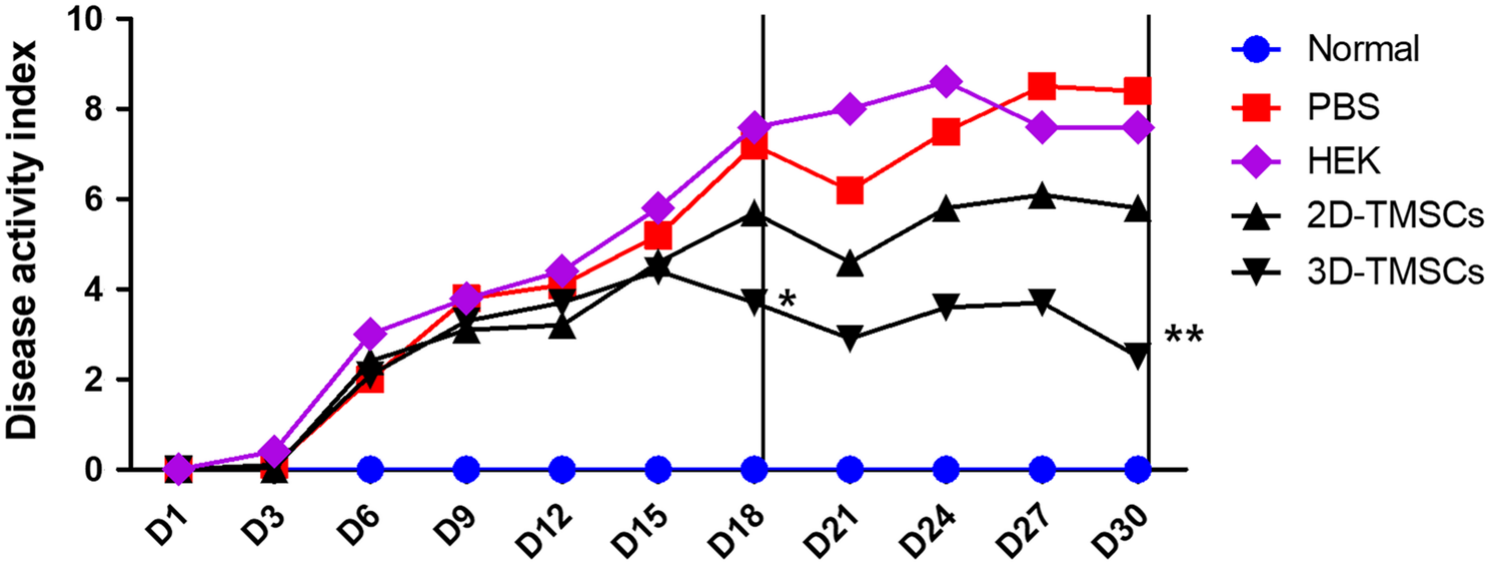
Disease activity index (DAI) analyzed after treatment with 3D-cultured TMSCs. The DAI scores based on stool consistency, fecal blood, and weight loss significantly decreased in the 3D-TMSC-treated group. *On day 18, the DAI scores in the 3D-TMSC-treated group were significantly lower than those in the DSS + PBS control (P < 0.001), DSS + HEK control (P = 0.013), and 2D-TMSC-treated groups (P = 0.022). **On day 30, the DAI scores in the 3D-TMSC-treated group were significantly lower than those in the DSS + PBS control (P = 0.001), DSS + HEK control (P = 0.010), and 2D-TMSC-treated groups (P = 0.004). 3D, three-dimensional; 2D, two-dimensional; TMSCs, tonsil-derived mesenchymal stem cells; DSS, dextran sulfate sodium; PBS, phosphate-buffered saline; HEK, human embryonic kidney.

**Figure 4:**
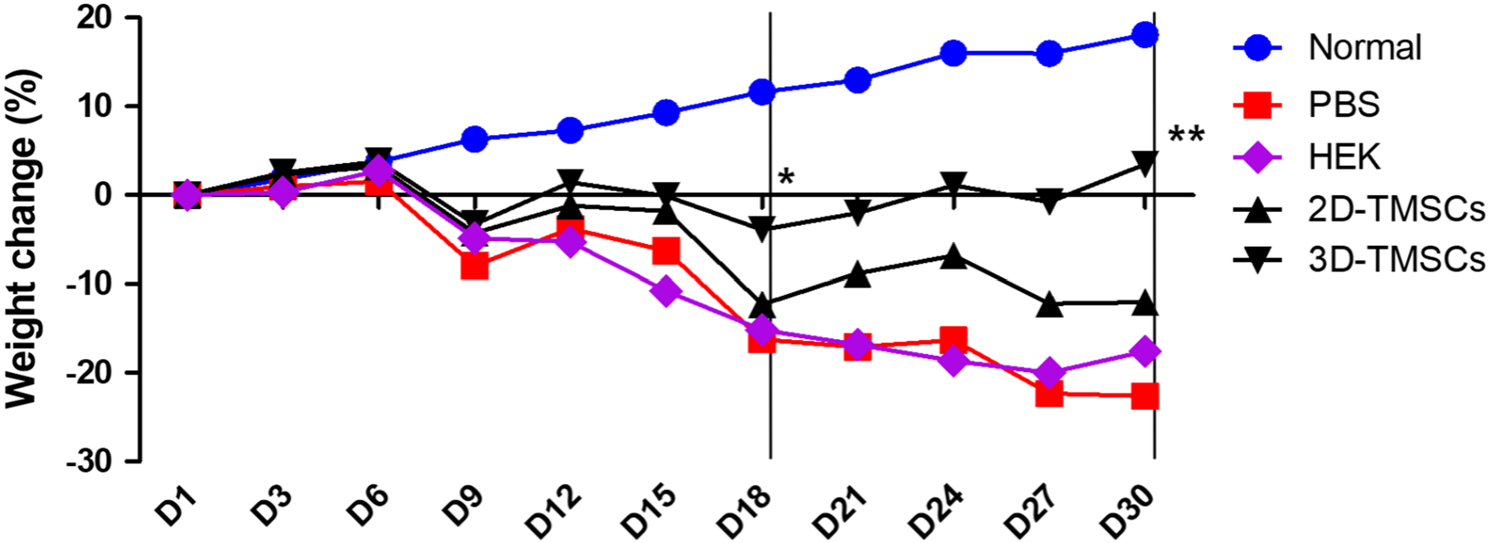
Weight recovery after treatment with 3D-cultured TMSCs. Body weight loss in the 3D-TMSC-treated group was significantly mitigated when compared with that in the DSS + PBS, the DSS + HEK and 2D-TMSC-treated groups. *On day 18, the body weight loss in the 3D-TMSC-treated group was lower than that in the DSS + PBS (P < 0.001), the DSS + HEK (P = 0.188), and 2D-TMSC-treated groups (P = 0.005). **On day 30, the body weight loss in the 3D-TMSC-treated group was significantly lower than that in the DSS + PBS (P = 0.001), the DSS + HEK (P = 0.027), and 2D-TMSC-treated groups (P = 0.005). 3D, three-dimensional; 2D, two-dimensional; TMSCs, tonsil-derived mesenchymal stem cells; DSS, dextran sulfate sodium; PBS, phosphate-buffered saline; HEK, human embryonic kidney.

We evaluated the therapeutic effects of 3D-cultured TMSCs in the DSS-induced chronic colitis murine model. All mice, except those belonging to the normal control group, exhibited bloody diarrhea on day 3 post-chronic colitis induction and body weight loss on day 6 post-chronic colitis induction. Macroscopic DAI scores based on stool consistency, body weight loss, and fecal blood in the 3D-TMSC-treated group were significantly lower than those in the DSS + PBS control group or DSS + HEK control group on days 18 and 30 post-chronic colitis induction [day 18, 3.7 ± 1.9 vs. 7.2 ± 2.2 (P < 0.001) or 7.6 ± 3.5 (P = 0.013); day 30, 2.5 ± 1.5 vs. 8.4 ± 3.8 (P < 0.001) or 7.6 ± 4.4 (P = 0.010); Fig. 3)]. Similarly, the DAI scores in the 3D-TMSC-treated group were significantly lower than those in the 2D-TMSC-treated group on days 18 and 30 post-chronic colitis induction [day 18, 3.7 ± 1.9 vs. 5.7 ± 2.7 (P = 0.022); day 30, 2.5 ± 1.5 vs. 5.8 ± 3.9 (P = 0.004); Fig. 3]. Additionally, the body weight loss (%) in the 3D-TMSC-treated group was significantly lower than that in the DSS + PBS control, DSS + HEK control and 2D-TMSC-treated groups on day 30 post-chronic colitis induction [+ 3.5 ± 12.6% vs. − 22.6 ± 15.5% (P < 0.001), − 17.6 ± 19.9% (P = 0.027), and − 12.0 ± 15.2% (P = 0.005); Fig. 4].

**Figure 5:**
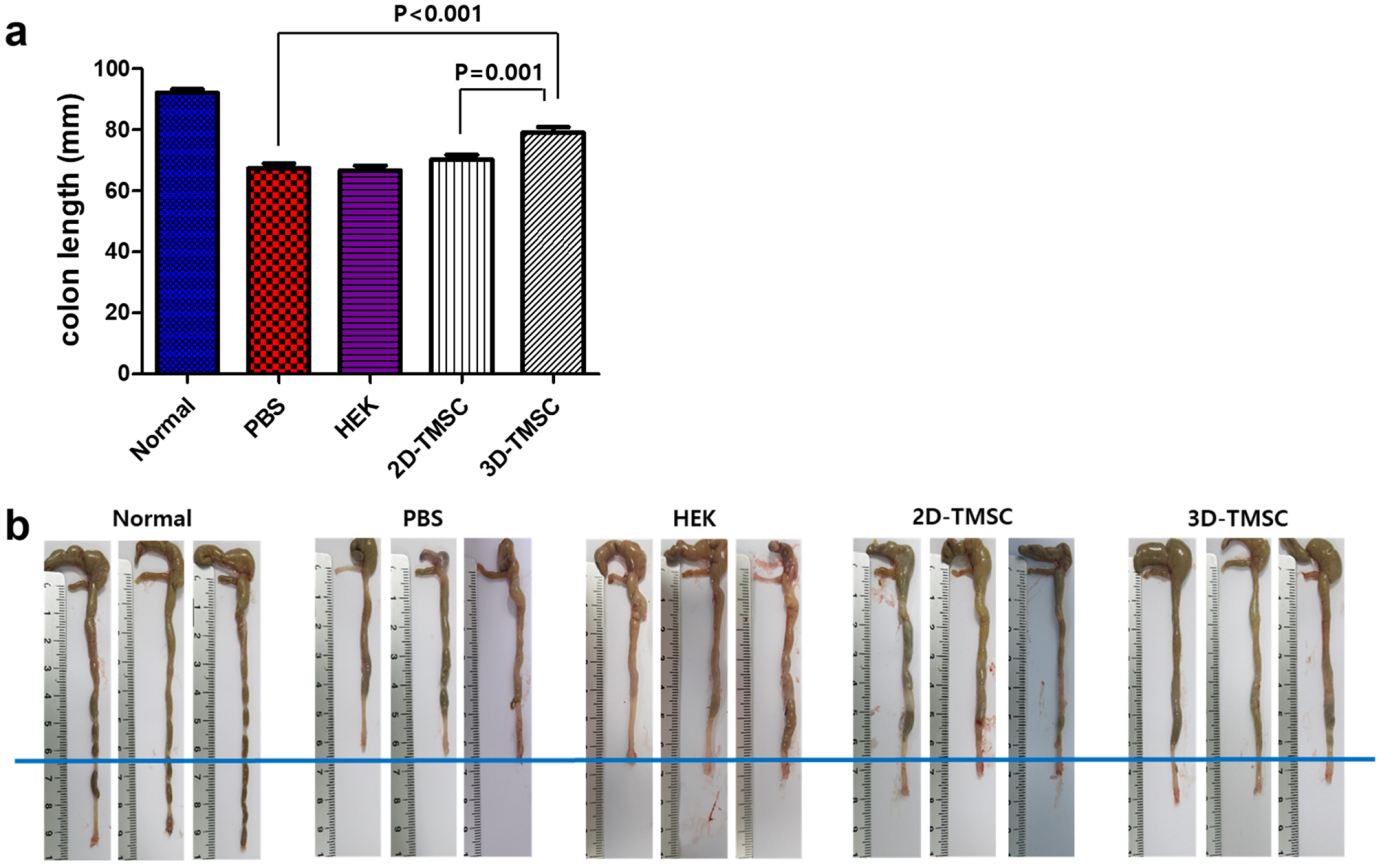
Colon length recovery after treatment with 3D-cultured TMSCs. (**a**) The colon length shortening in the 3D-TMSC-treated group was significantly lower than that in the DSS + PBS control (P < 0.001), DSS + HEK control (P = 0.001) and 2D-TMSC-treated groups (P = 0.001). (**b**) The lengths of the colon in each group are indicated. 3D, three-dimensional; 2D, two-dimensional; TMSCs, tonsil-derived mesenchymal stem cells; DSS, dextran sulfate sodium; PBS, phosphate-buffered saline.

Furthermore, colitis-associated colon length shortening in the 3D-TMSC-treated group (79.1 ± 7.5 mm) was significantly lower than that in the DSS + PBS control (67.1 ± 5.5 mm) and DSS + HEK control groups (66.0 ± 2.2 mm) (P < 0.005 for all; Fig. 5). The recovery of colon shortening in the 3D-TMSC-treated group (79.1 ± 7.5 mm) was also significantly higher than that in the 2D-TMSC-treated group (70.3 ± 6.2 mm; P = 0.001; Fig. 5).

#### Cytokine expression in the colonic tissues of mice belonging to the 3D-TMSC-treated group

**Figure 6:**
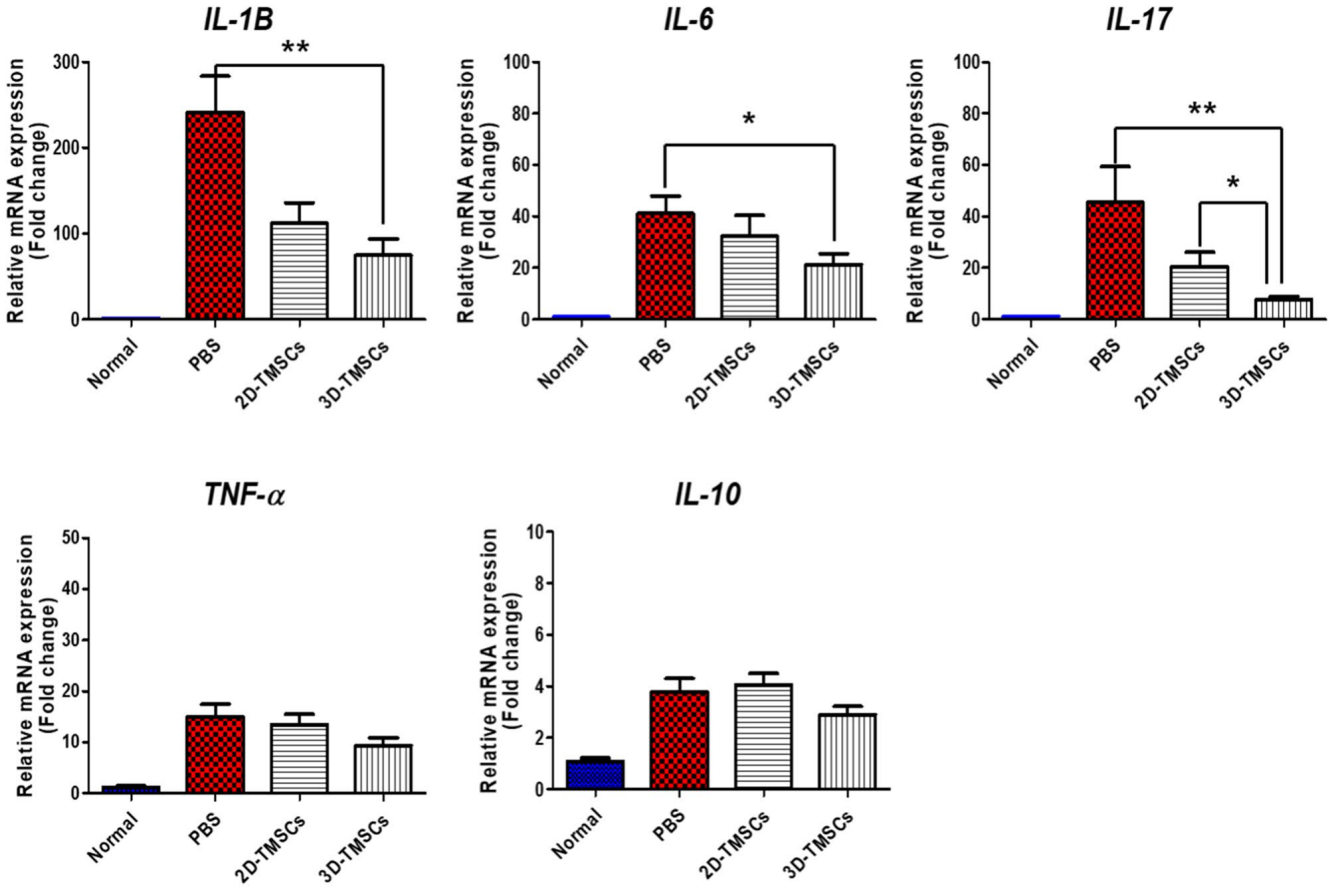
mRNA expression of cytokines in colon tissue treated with 3D-TMSC-treated groups. *IL-1β, IL-6, and IL-17* levels were significantly lower in the 3D-TMSC-treated group compared with the colitis control group. *P < 0.05, **P < 0.005. 3D, three-dimensional; TMSCs, tonsil-derived mesenchymal stem cells; IL, interleukin; PBS, phosphate-buffered saline; TNF-α, tumor necrosis factor α.

**Figure 7:**
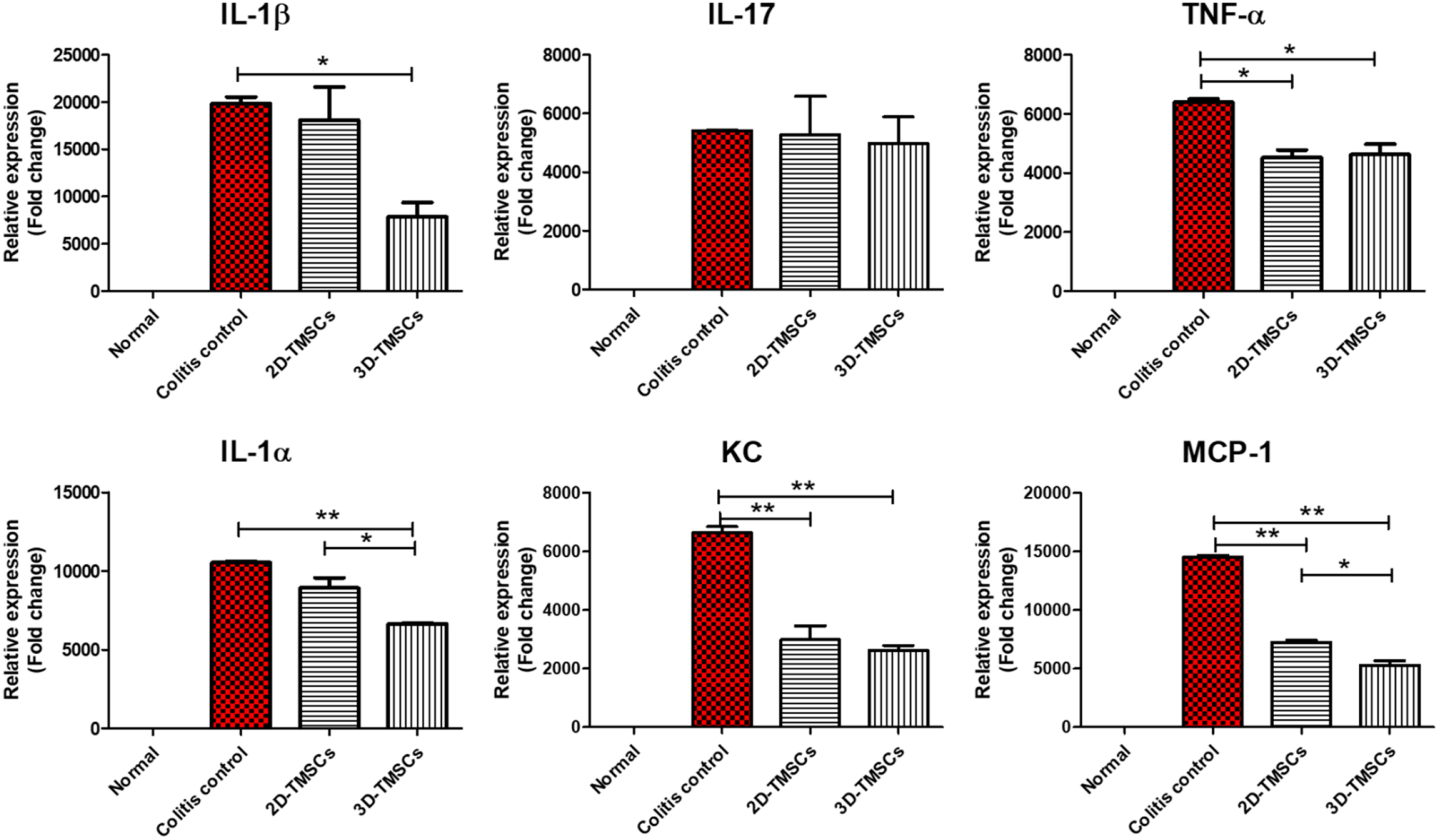
Cytokine analysis in the colon tissues of the 3D-TMSC-treated group. The levels of other pro-inflammatory cytokines, including IL-1β, TNF-α, and IL-1α, and chemokines associated with immune-cell activation, such as KC and MCP-1 were significantly downregulated in the 3D-TMSC-treated group. *P < 0.05, **P < 0.005. 3D, three-dimensional; TMSCs, tonsil-derived mesenchymal stem cells; IL, interleukin; TNF-α, tumor necrosis factor α; KC, keratinocyte chemoattractant; MCP-1, monocyte chemoattractant protein-1.

We measured the mRNA expression levels of inflammatory cytokines in the colon tissues of mice in each group. The mRNA expression levels of pro-inflammatory cytokines, including *IL-1B, IL-17,* and *IL-6*, were significantly downregulated in the 3D-TMSC-treated group when compared with those in the colitis control group (P = 0.001, P = 0.001, and P = 0.024, respectively, Fig. 6). Although *IL-1B, IL-17*, and *IL-6* levels were lower in the 3D-TMSC-treated group than in the 2D-TMSC-treated group, the difference was not statistically significant, except for *IL-17* (P = 0.036, Fig. 6). We also measured the protein expression levels of cytokines in the colon tissue using a cytokine array (Fig. 7). Consistent with the downregulated mRNA levels of *IL-1B*, the protein levels of IL-1β were significantly downregulated in the 3D-TMSC-treated group. However, the level of IL-17 was not significantly different between the groups. The levels of other pro-inflammatory cytokines, including TNF-α and IL-1α, and chemokines associated with immune cell activation, such as keratinocyte chemoattractant (KC; CXCL-1) and monocyte chemoattractant protein-1 (MCP-1) were significantly downregulated on treatment with 3D-TMSCs.

#### Histopathological improvement after 3D-cultured TMSCs treatment

**Figure 8:**
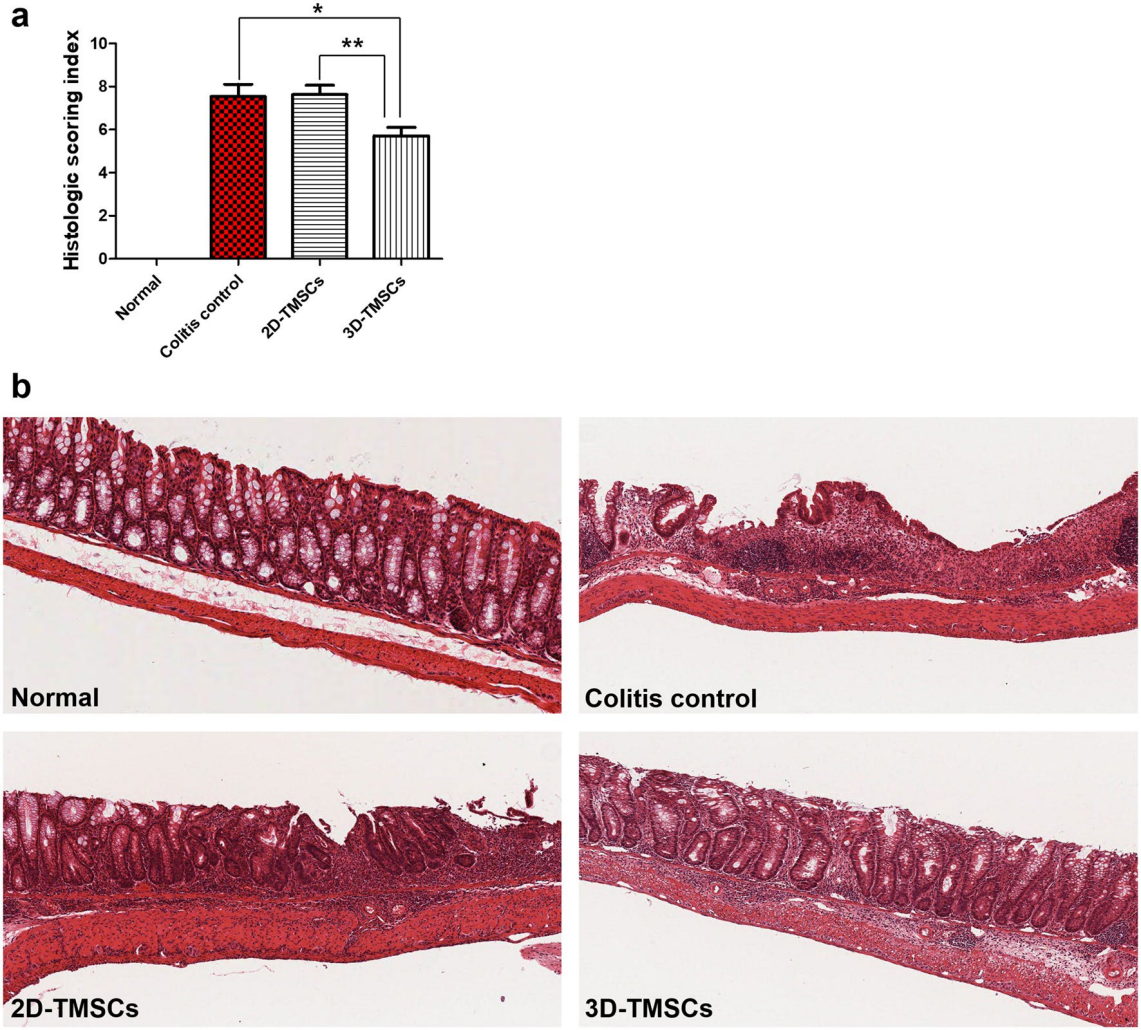
Histopathological recovery after treatment with 3D-cultured TMSCs. (**a**) Histopathological scoring index showed significant recovery in the 3D-TMSC-treated group when compared with the colitis control. (**b**) Histological results of colon in each group. [Hematoxylin–eosin (H&E) stain, ×100] *P < 0.05, **P < 0.005. 3D, three-dimensional; TMSCs, tonsil-derived mesenchymal stem cells; PBS, phosphate-buffered saline.

As shown in Fig. 8, the colonic structure in the DSS-induced chronic colitis model was disrupted and characterized by the loss of crypts, diffuse mucosal and submucosal edema, infiltration of mononuclear cells, and ulcerations. The histological scoring index (HSI) values, which indicate the severity and extent of inflammation and crypt damage, in the 3D-TMSC-treated group (7.5 ± 2.8) were significantly lower than those in the colitis control group (5.7 ± 2.8; P = 0.01; Fig. 8). Histological analysis revealed that treatment with 3D-cultured TMSCs mitigated the DSS-induced severe inflammatory cell infiltration, loss of crypts, and ulceration (Fig. 8). The HSI values in the 3D-TMSC-treated group (5.7 ± 2.8) were also significantly lower than those in the 2D-TMSC-treated group (7.6 ± 2.7; P = 0.002).

#### TMSC localization in the peritoneum of mice using immunofluorescence

**Figure 9:**
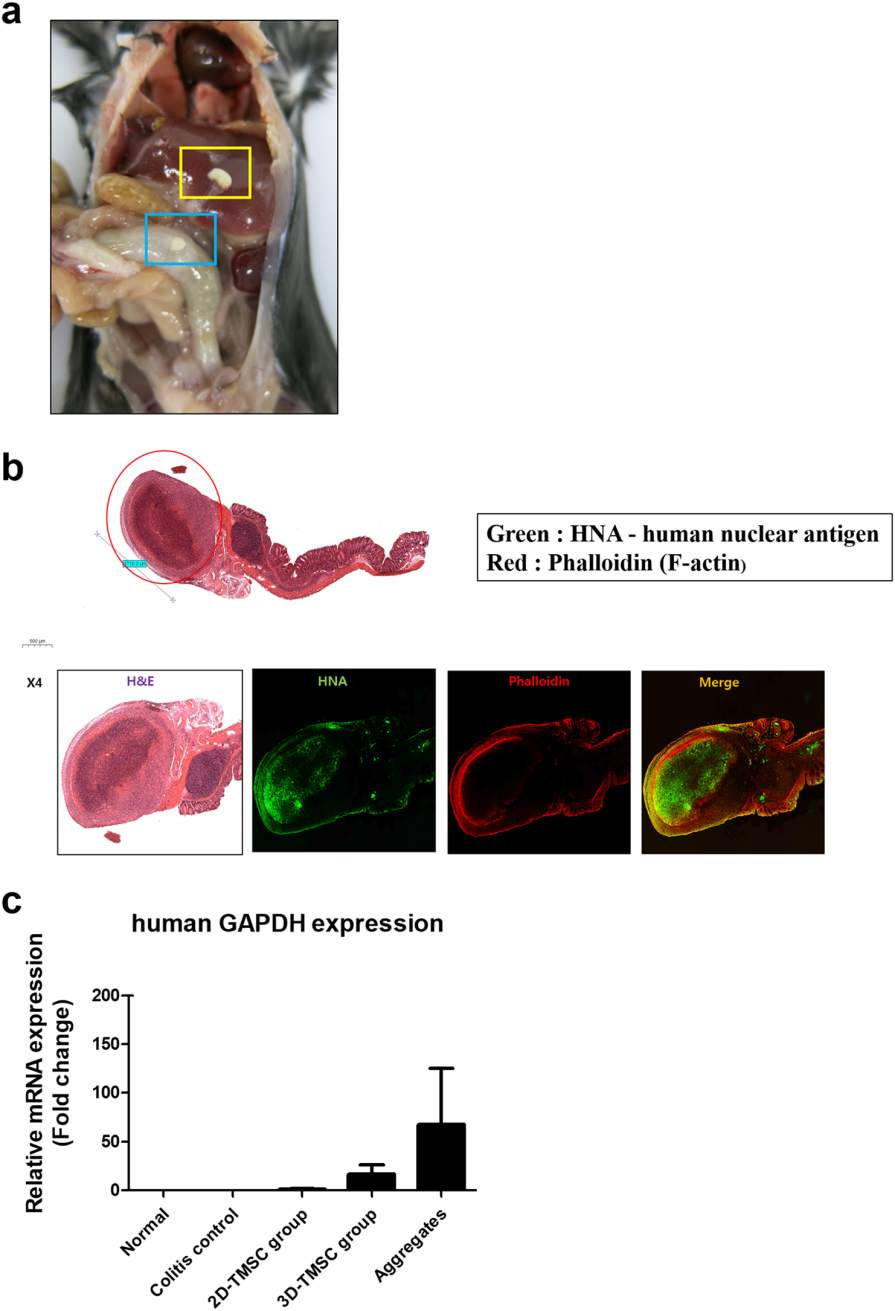
Localization of 3D-cultured TMSCs in the peritoneum of mice analyzed using immunofluorescence staining. (**a**) Image of the gross morphology of aggregates in the peritoneum of the mouse. (**b**) One aggregate was adjacent to the mouse colon. Fluorescence microscopy revealed that the previously observed aggregate was stained with green-colored anti-human nuclear antigen antibody. (**c**) The levels of human DNA in the peritoneal lavage fluid of each groups and aggregates. 3D, three-dimensional; TMSCs, tonsil-derived mesenchymal stem cells; H&E, hematoxylin–eosin; HNA, human nuclear antigen.

White spherical aggregates were observed in the euthanized mice. We assumed that the injected TMSCs formed an aggregate in the peritoneal space of mice. Some aggregates were adjacent to the mouse intestine, whereas other aggregates were not attached to any organ and were observed in the omentum and mesentery (Fig. 9a). Spheroids were observed in the 3D-TMSC-treated group but not in the 2D-TMSC-treated group. To confirm the location of TMSCs in vivo, the cells were tracked with anti-human nuclear antigen antibodies using immunofluorescence. Fluorescence microscopy analysis revealed that the previously observed aggregates were stained with green-colored anti-human nuclear antigen antibodies, which indicated that the transplanted 3D-cultured TMSCs formed a cluster in the peritoneum irrespective of colon inflammation (Fig. 9b). In addition, the expression levels of human DNA in the peritoneal lavage fluid of each groups and aggregates were measured. The expression levels of human DNA were the highest in the aggregates, followed by the peritoneal lavage fluid of 3D-TMSC-treated and 2D-TMSC-treated groups (Fig. 9c). This indicates that these whitish aggregates in the mouse peritoneal cavity comprise TMSCs.

## Discussion

In the present study, we evaluated the therapeutic effects of 3D-cultured TMSCs and compared them with those of 2D-cultured TMSCs in a chronic murine colitis model. 3D-cultured TMSC treatment ameliorated the clinical symptoms, including fecal blood, body weight loss, and shortening of colon length in a chronic murine colitis model. Additionally, histological improvement was achieved with 3D-cultured TMSCs, which was not observed through 2D-cultured TMSC treatment. Moreover, we showed that the 3D-culture method could enhance the expression of anti-inflammatory cytokines and growth factors in TMSCs and the survival of TMSCs in vivo after transplantation.

Initially, MSCs were thought to infiltrate into inflamed tissues and engage in tissue regeneration by differentiating into mature intestinal epithelial cells^18^. However, recently published studies showed that the paracrine effect is a more important mechanism of MSCs irrespective of their location in vivo^19^. Although MSCs are considered as a promising new therapeutic modality in various diseases, including IBD, clinical trials showed controversial outcomes^20^. Reduced cell viability of MSCs after in vivo transplantation has limited its usability. Moreover, to maintain effective paracrine activity of MSCs, the transplanted cells should endure harsh microenvironments (e.g., oxidative, inflammatory, and hypoxic conditions), which reduce the rate of cell engraftment and survival^21^. Recently, 3D-culture method has been evaluated as a modality to improve the efficacy of MSC therapy. More recently, organoid culture methods, which are physiologically more relevant, have been developed to overcome the shortcomings of MSC therapy^22^.

In our previous studies, we evaluated the therapeutic effect of TMSCs in acute and chronic colitis models^14,15,23^. IP injection of TMSCs significantly ameliorated DSS-induced colitis; however, no histologically significant effects were observed. Additionally, in acute and chronic colitis models, TMSC-conditioned medium showed similar therapeutic efficacy as TMSCs. However, histological improvements were not achieved through treatment with TMSC-conditioned medium. Moreover, we tried to improve the therapeutic efficacy of TMSCs pretreated with ascorbic acid and metformin, however, we could not achieve the desired results (data not shown). In the current study, the 3D-culture method showed significant enhancement in the therapeutic efficacy of TMSCs in addition to histological improvement.

The efficacy of 3D-culture method for MSC therapy has been evaluated for several disease models, including those of inflammation, ischemic injury, and cancer^21,24–26^. In a previous study, 3D-cultured ADSCs showed enhanced inhibitory effects on liver cancer cells when compared with 2D-cultured or sphere-cultured ADSCs^26^. Xu et al. reported that 3D-culture method enhanced cell survival and paracrine effect of MSCs, thereby showing therapeutic effects on acute kidney injury model^12^. Additionally, a recently published study demonstrated that this enhanced cell function and cell viability of MSCs by 3D-culture method are mediated via decreased reactive oxygen species production and autophagy activation^21^. Molendijk et al. investigated the therapeutic effect of intraluminally-injected 3D-cultured bone marrow-derived MSCs (BMSCs) in a DSS-induced acute colitis model^27^. However, this study only evaluated the therapeutic effect of 3D-cultured BMSCs, administered through limited injection methods, in an acute colitis model, and comparison with 2D-cultured MSCs was not appropriately performed^27^. In the current study, we evaluated enhanced therapeutic effects in a chronic colitis model, which are clinically similar to chronic human IBD.

Enhanced cell survival of 3D-cultured TMSCs was demonstrated in our study, which corresponds with the results obtained in the previous studies. Upon tracking the location of transplanted 2D- and 3D-cultured TMSCs on day 31, 3D-cultured TMSCs, which were injected at least 14 days before euthanizing the mice, were observed to form aggregates in the peritoneum independently of the inflamed colon, however, this effect was not observed upon 2D-cultured TMSC administration. Interestingly, the transplanted 3D-cultured TMSCs were detected in the peritoneum, but not incorporated in the colon tissues, implying that the therapeutic effects of 3D-cultured TMSCs were not caused by gut-homing. Our results are in accordance with a previous study by Sala et al., who reported that intraperitoneally injected BMSCs do not localize in the intestine, instead they form aggregates in the peritoneum and produce cytokines^19^. The mechanisms underlying the survival of transplanted human 3D-TMSCs in immunocompetent mice have not been elucidated. However, long-term survival of human MSCs in mice has also been reported in previous studies. Dhada et al. examined the viability of MSCs using the nanoprobe method and reported that 5% of transplanted human MSCs were detected in mice at day 10 post-transplantation^28^. Ning et al. tracked the labeled human MSCs in the mouse pulmonary fibrosis injury model for up to day 23 post-transplantation using CT^29^. Although spontaneous cell death occurs after the transplantation of 3D-TMSCs, this process may be delayed by the immunomodulatory function of 3D-TMSCs, which is associated with the secretion of immune-modulatory cytokines, including *IDO-1, TSG-6, and transforming growth factor β (TGF-β)*. In addition, the expression level of *CXCR4* in the 3D-cultured TMSCs was higher than that in the 2D-cultured TMSCs. *CXCR4* is reported to increase the viability and migration of MSCs^30,31^. Therefore, *CXCR4* may play a critical role in the enhanced survival of 3D-TMSCs.

Paracrine secretion of therapeutic cytokines, not tissue regeneration by gut-homing, may play an important role in the treatment mechanism of TMSCs. Although the exact mechanism of 3D-cultured TMSC-mediated attenuation of chronic murine colitis is unclear, enhanced paracrine secretion of these beneficial cytokines may be involved in imparting this effect. In our study, the expression of anti-inflammatory cytokines, including *IL-10 and TSG-6,* was significantly increased via 3D-culture method (P < 0.05). In a previous study, TSG-6 was reported to be a key cytokine in MSC therapy and promoted the expansion of regulatory macrophages that expressed IL-10 and inducible nitric oxide synthase, and reduced serum levels of interferon-6, IL-6, and TNF-α^19^. Moreover, in experimental myocardial infarction model, treatment with MSCs reduced tissue damage by producing TSG-6^32^. Importantly, TMSCs highly expressing TSG-6 showed therapeutic effect in an acute graft-versus-host disease (GVHD) mouse model^33^. In our study, *TSG-6* production was markedly increased by 3D-culture method, thereby, enhancing the therapeutic effect of TMSCs. Moreover, the expression levels of cytokines associated with immunomodulatory effects, including *TGF-β and IDO-1,* were significantly upregulated in the 3D-cultured TMSCs. Additionally, the expression levels of *IL-1β, IL-6, and IL-17* were downregulated in the colon tissues of the 3D-TMSC-treated group, which may be due to the production of cytokines associated with immune-regulatory functions, such as *TSG-6, TGF-β, and IDO-1.* The upregulated expression of *VEGF* in the 3D-cultured TMSCs may also promote the repair of damaged intestinal mucosa by enhancing TMSC migration and proliferation and accelerating the growth of vessels^34^.

In summary, the present study demonstrated that 3D-cultured TMSCs significantly ameliorated chronic colitis by reducing clinical symptoms, recovery of colon shortening, and histological improvements. We further demonstrated enhanced survival of 3D-cultured TMSCs in the peritoneum. Additionally, we demonstrated that the increased paracrine effect of anti-inflammatory cytokines, including *IL-10, TSG-6, TGF-β, and IDO-1,* may play a critical role in mediating the therapeutic effect of 3D-cultured TMSCs. Thus, the 3D-culture method provided a novel approach to enhance TMSC function and, thereby, could have therapeutic applications in IBD.

## Methods

### Isolation and expansion of TMSCs

Subjects aged less than 18 years were recruited in this study. Written informed consent was obtained from a parent and/or patients’ legal guardians for studies on tonsil tissues. This study was approved by the Ewha Womans University Medical Center institutional review board (ECT 11–53-02). All experiments were performed in accordance with the institutional ethical guidelines and the Declaration of Helsinki. All tonsil tissues used in this study were obtained from a single donor. TMSCs were isolated and cultured as described in our previous studies^15,35^. Briefly, tonsil tissues were obtained during tonsillectomy in patients younger than 10 years of age. The tonsil tissues were minced and digested in RPMI 1640 medium (Invitrogen, Carlsbad, CA, USA) supplemented with 210 U/mL collagenase type I (Invitrogen) and 10 g/mL DNase (Sigma-Aldrich, St. Louis, MO, USA) at 37 °C for 30 min. Digested tissues were washed using Dulbecco’s modified Eagle’s medium–high glucose (DMEM-HG; Welgene, Daegu, South Korea) supplemented with 20% fetal bovine serum (FBS; Invitrogen), and washed again using DMEM-HG supplemented with 10% FBS. From the prepared tonsil tissues, we isolated mononuclear cells using Ficoll-Paque (GE Healthcare, Little Chalfont, UK) density gradient centrifugation. The isolated mononuclear cells were then cultured in cell culture plates, and non-adherent cells were removed after 8 h of seeding. The remaining adherent cells were further cultured for 2 weeks and passaged. The passaged cells (hereafter referred to as TMSCs) were stored in liquid nitrogen for future experiments. The MSC characteristics of TMSCs according to the minimal criteria for defining multipotent mesenchymal stromal cells^36^ were confirmed in our previous study^15^ and other studies^37^. In brief, the immunophenotypic surface marker assay results revealed that TMSCs were negative for hematopoietic cell markers, such as CD14, CD34, and CD45 and positive for common MSC markers, such as CD73, CD90, and CD105. In addition, cell-specific staining assays were performed to examine the differentiation of TMSCs into adipocytes, chondrocytes, and osteoblasts. TMSCs could differentiate into these three cell types upon induction with commercially available differentiation media^15^.

### Induction of chronic colitis using DSS in mice

The animal models used for the experiment were 7-week-old C57BL/6 male mice (Orient Bio Co., Ltd., Sungnam, Gyeonggi, Korea) with an average weight of 20–22 g. The mice were acclimatized for 7 days in a standardized environment at the facility of the Ewha Womans University Medical Research Institute prior to the experiment. Day and night conditions were provided at 12 h intervals, and temperature (23 ± 2 °C) and humidity (45–55%) were set to appropriate levels. Experiment and procedures were approved and all experiments were performed in accordance with experimental research protocol approved by the Ethics Committee for Animal Research of Ewha Womans University (EUM19-0464, ESM18-0415). This study was performed according to the standards articulated in the ARRIVE guidelines^38^. Chronic colitis in mice was induced by oral administration of 1.5% DSS (MP biochemical, Irvine, CA, USA) for 5 days, followed by an additional 5 days of tap water feeding; overall 3 such cycles (total 30 days) were performed. We used PBS or human embryonic kidney 293 (HEK293) cells as a sham control for colitis.

### 3D-culture of TMSCs

3D-cultured spheroids of TMSCs were formed using StemFIT 3D micro 853 wells (Microfit, Hanamsi, Korea). One plate had 853 wells, each of 400 μm in size, and 1.0 × 10^6^ cells/mL were cultured in a plate. Therefore, each spheroid comprised approximately 1200 TMSCs. TMSCs from passages 6 to 8 were used to form spheroids. After culturing, spheroids with a size of approximately 200 μm were formed. The expression levels of Nanog, Sox2, and Oct4 (stem cell markers) in the 3D-cultured TMSCs were comparable to those in the 2D-cultured TMSCs. This suggests that 3D-cultured TMSCs maintained the MSC phenotype (Supplementary Figure 2). We used 3D-cultured TMSCs on day 1 of spheroid formation. The size of the 3D-cultured TMSCs was measured on days 1, 2, and 3. The qRT-PCR analysis was performed to determine the expression levels of *IL-4, IL-5, IL-10, CXCR4, SDF-1, TGF-β, IDO-1, α-1 chain of type 1 collagen (COL1A1), VEGF, B-cell CLL/lymphoma 2 (Bcl-2), Bcl-2 associated X (Bax), and TSG-6* in TMSC lysates using the QuantStudio 3 real-time PCR system (Applied Biosystems, Waltham, MA, USA) (Supplementary Table 1). Detailed procedures are described in Supplementary Methods.

### Experimental design

In the chronic colitis model, mice were randomly assigned to five groups: (1) normal control (n = 10), (2) DSS + PBS control (n = 17), (3) DSS + HEK control (n = 5), (4) 2D-TMSC-treated group (n = 17), and (5) 3D-TMSC-treated group (n = 18). The DSS + PBS group was intraperitoneally injected with phosphate-buffered saline (PBS) on days 6 and 16 post-chronic colitis induction. The DSS + HEK control group was intraperitoneally injected with HEK293 cells (1 × 10^6^ cells) on days 6 and 16 post-chronic colitis induction. Meanwhile, the 2D-TMSC-treated and 3D-TMSC-treated groups were intraperitoneally injected with 1.0 × 10^6^ TMSCs/500 μL PBS on days 6 and 16 post-chronic colitis induction. We used StemFIT 3D micro 853 wells, and each spheroid comprised approximately 1200 TMSCs. Therefore, the 3D-TMSC-treated group was intraperitoneally injected with 853 spheroids.

### Assessment of therapeutic effect of TMSCs

#### DAI scoring

For each group, the body weight and DAI scores, including weight change, stool consistency, and occult or fecal blood, were determined as previously reported (Supplementary Table 2)^39^.

#### Measuring colon length and histopathological scoring

On day 31 of chronic colitis induction, mice were euthanized with CO_2_ gas inhalation, and colon specimens were acquired from the proximal and distal parts of the dissected colon and fixed with 10% formalin, followed by paraffin sectioning and hematoxylin–eosin (H&E) staining. Two specimens from each proximal and distal colon were evaluated using a previously reported histological colitis scoring system (Supplementary Table 3)^40^. The severity and extent of inflammation, the level of crypt damage, and the damaged portion (%) of the whole colon were evaluated. Finally, the averages of the HSI values were compared between the groups.

#### Quantitative reverse transcriptase polymerase chain reaction (qRT-PCR) for cytokine expression

qRT-PCR for determining the expression of pro-inflammatory cytokines, namely, *IL-1β, IL-6, TNF-α, IL-17*, and the anti-inflammatory cytokine *IL-10* in colonic tissues was performed using the QuantStudio 3 real-time PCR system (Applied Biosystems, Waltham, MA, USA). Detailed procedures are described in the Supplementary methods.

#### Cytokine array analysis

The levels of cytokines in the colonic tissue were analyzed using the Proteome Profiler Mouse Cytokine Array Panel A kit (R&D Systems, Minneapolis, MN, USA), following the manufacturer’s instructions. In brief, the tissues were lysed using radioimmunoprecipitation assay buffer (containing protease inhibitor cocktail). The protein concentration in the lysate was measured using a BCA Protein assay kit (Thermo Scientific, Waltham, MA, USA). Tissue protein diluted in array buffer was incubated with the ready-to-use pre-coated array membranes overnight at 4 °C on a rocking platform shaker. The membrane was washed and incubated with streptavidin–horseradish peroxidase (HRP) buffer for 30 min. Next, the membrane was washed and incubated with the Chemi Reagent mixture at 23–27 °C for 1 min. The membrane was analyzed using the LAS-300 system (Fujifilm, Tokyo, Japan). Dot density was analyzed using Multi Gauge 3.0.

#### Immunofluorescence analysis for visualizing TMSC localization and determination of human DNA expression levels in the mouse peritoneal cavity cells

Colon specimens were fixed as paraffin blocks and 4 μm wide sections were resected. After deparaffinization and hydration, the specimens were blocked with mouse IgG reagent using a mouse on mouse (M. O. M) kit (Vector Laboratories, CA, USA). TMSCs were then stained with human nuclear antigen monoclonal antibody (MyBioSource, CA, USA). After fluorescence staining with Avidin DCS (Vector Laboratories, CA, USA), the specimens were mounted with Vectashield phalloidin (Vector Laboratories, CA, USA), and observed using fluorescence microscopy.

The expression levels of human DNA in the peritoneal lavage fluid of each treated groups and aggregates were measured. The mice were euthanized and sprayed with 70% ethanol. The outer skin of the peritoneum was dissected using scissors and forceps and gently pulled back to expose the inner skin surrounding the abdominal cavity. The peritoneal lavage fluid was collected by injecting 5 mL of ice-cold saline into the peritoneal cavity using a 26 G needle. The collected cell suspension was centrifuged at 1500 rpm for 8 min. The supernatant was discarded and the cell pellet was stored at − 70 °C. Total RNA was extracted from the isolated intraperitoneal cells using TRIzol Reagent (Ambion, Life Technologies, Carlsbad, CA, USA). cDNA was synthesized using the Moloney murine leukemia virus reverse transcriptase (M-MLV RT) kit (Promega, Fitchburg, WI, USA). The qRT-PCR analysis was performed using the human GAPDH-specific primer set (Forward 5′-TCAAGGCTGAGAACGGGAAG-3′ and Reverse 5′-CGCCCCACTTGATTTTGGAG-3′) to confirm the presence of human-origin cells among intraperitoneal cells.

### Statistical analysis

The measured values of all experimental results are expressed as mean ± standard deviation (s.d.). Comparisons between two groups, including parametric or non-parametric analyses, were performed using an unpaired Student’s *t*-test or Mann–Whitney U test, respectively. P values less than 0.05 were considered statistically significant. All statistical analyses were performed using IBM SPSS version 22.0 (IBM Corp., Armonk, NY, USA).

## Supplementary Information

Supplementary Information.

## Data Availability

The raw data supporting the conclusions of this article will be made available by the corresponding author, without undue reservation, to any qualified researcher.
