# Supplementary material for: Three-dimensional culture method enhances the therapeutic efficacies of tonsil-derived mesenchymal stem cells in murine chronic colitis model
Source: Sci Rep. 2021 Oct 1;11:19589. doi: 10.1038/s41598-021-98711-4 (PMC8486762; doi:10.1038/s41598-021-98711-4)

**Three-Dimensional Culture Method Enhances the Therapeutic Efficacies of Tonsil-derived Mesenchymal Stem Cells in Murine Chronic Colitis Model**

Eun Mi Song^1^, Yang Hee Joo^1^, A Reum Choe^1^, Yehyun Park^1^, Chung Hyun Tae^1^, Ji Teak Hong^1^, Chang Mo Moon^1^, Seong-Eun Kim^1^, Hye-Kyung Jung^1^, Ki-Nam Shim^1^, Kyung-Ah Cho^2^, Inho Jo^3^, and Sung-Ae Jung^1*^

^1^Department of Internal Medicine, College of Medicine, Ewha Womans University, Seoul, Korea

^2^Department of Microbiology, College of Medicine, Ewha Womans University, Seoul, Korea

^3^Department of Molecular Medicine, College of Medicine, Ewha Womans University, Seoul, Korea

*Corresponding author:

Sung-Ae Jung, MD, PhD.

Departments of Internal Medicine, College of Medicine, Ewha Womans University, Seoul, Korea

E-mail: jassa@ewha.ac.kr

Tel: 02-6986-1620

**Supplementary materials**

**Supplementary Methods**

**Quantitative reverse transcriptase polymerase chain reaction (qRT-PCR) for cytokine expression**

The colon specimen was stored at −70 °C in a cryogenic freezer until analysis. The 2D-cultured and 3D-cultured TMSCs and the colon specimens were prepared for qRT-PCR analysis using the same method. The 2D-TMSCs and 3D-TMSCs or the colon specimen were lysed with 1 mL of TRIzol Reagent (Ambion, Life Technologies, Carlsbad, CA, USA). The lysate was then incubated with 200 μL of chloroform (Sigma) and vortexed. After leaving the sample at the room temperature for 2 to 3 min, it was centrifuged at 13,000 ×g (4℃) for 10 min. The supernatants were added into a new tube and the same amount of isopropanol (Sigma) was added; after mixing by inversion, the sample was left at room temperature for 5 min, and then centrifuged again at 13,000 ×g (4℃) for 10 min. The cell pellet was washed with 75% ethanol for 2–3 times and dried at room temperature. Nuclease-free water was then added, and the RNA concentration was measured with Nabi (nano-drop) (Micro Digital, Korea). Then, 2 μg of RNA and 0.5 μg of oligo dT primer were mixed, and the sample was left at 70℃ for 10 min. Next, 200 units of Molony Murine Leukemia Virus Reverse Transcriptase (M-MLV RT) (Promega, Fitchburg, WI, USA), 25 units of rRNasin Ribonuclase inhibitor (Promega), 5 X RT buffer and 2 mM of dNTP were added, and the final volume was set to 25 μL by adding an appropriate amount of nuclease-free water. They were treated at 42℃ for 60 min, and 95℃ for 5 min, and then stored at 4℃. Quantstudio 3 real-time polymerase chain reaction (PCR) system (Applied Biosystems, Waltham, MA, USA) was used for analysis, using 0.1 μg of the synthesized cDNA as a template for the 2X Power SYBR Green PCR Master mix (Applied Biosystems) and each primer set (Supplementary Table 4). The primers used in this study were made by Macrogen (Korea). Pro-inflammatory cytokines, interleukin (IL)-1β, IL-6, tumor necrosis factor α (TNFα), IL-17, and the anti-inflammatory cytokine IL-10 levels were measured; each primer sequence is shown in Supplementary Table 3. Each PCR was performed after 10 min of pre-denaturation at 95℃, 15 seconds at 95℃ and 1 min at 60℃; this was repeated 40 times. After the PCR was complete, a melting curve was drawn to check the accuracy of gene amplification. For the internal compensation of gene expression level, the house keeping gene, glyceraldehyde-3-phosphate dehydrogenase (GAPDH), was also used, and relative gene expression levels were presented as 2-ΔΔCt values.

**Supplementary Table 1. Primer sequences used for qRT-PCR (quantitative real-time polymerase chain reaction) analysis of TMSCs.**

| **Primer** |  | **Sequence** |
| --- | --- | --- |
| ***Nanog*** | Forward | 5′-AAT ACC TCA GCC TCC AGC AGA T-3′ |
|  | Reverse | 5′-TGC GTC ACA CCA TTG CTA TTC TT-3′ |
| ***Oct4*** | Forward | 5′-CTT GCT GCA GAA GTG GGT GGA A-3′ |
|  | Reverse | 5′-CTG CAG TGT GGG TTT CGG GCA-3′ |
| ***SOX9*** | Forward | 5′-AGA CCA GTA CCC GCA TCT-3′ |
|  | Reverse | 5′-CGC TCC GCC TCC AC-3′ |
| ***CXCR4*** | Forward | 5′-GCA TGA CGG ACA AGT ACA GGC T-3′ |
|  | Reverse | 5′-AAA GTA CCA GTT TGC CAC GGC-3′ |
| **SDF-1** | Forward | 5′-ACA CTC CAA ACT GTG CCC TTC-3′ |
|  | Reverse | 5′-CCA CGT CTT TGC CCT TTC ATC-3′ |
| ***COL1A1*** | Forward | 5′-GCT GAA TGC CAA TGT GGT T-3′ |
|  | Reverse | 5′-CCA GTC AGA GTG GCA CAT CTT G-3′ |
| ***VEGF*** | Forward | 5′-ACG ATC GAT ACA GAA ACC ACG-3′ |
|  | Reverse | 5′-CTC TGC GCA GAG TCT CCT CT-3′ |
| **Bcl-2** | Forward | 5′-ATG TGT GTG GAG AGC GTC AA-3′ |
|  | Reverse | 5′-CAG GAG AAA TCA AAC AGA GGC-3′ |
| ***Bax*** | Forward | 5′-CCC GAG AGG TCT TTT TCC GAG-3′ |
|  | Reverse | 5′-CCA GCC CAT GAT GGT TCT GAT-3′ |
| ***TGF-β*** | Forward | 5′-GTG ACA GCA GG ATA ACA CAC TG-3′ |
|  | Reverse | 5′-CAT GAA TGG CCA GGT C-3′ |
| ***IDO-1*** | Forward | 5′-GCC CTT CAA GTG TTT CAC CAA-3′ |
|  | Reverse | 5′-CCA GC AGA CAA ATA TAT GCG A-3′ |
| **IL-4** | Forward | 5′-GAC GCT GAT CCG ATT CCT GAA A-3′ |
|  | Reverse | 5′-TTC CAA CGT ACT CTG GTT GGC T-3′ |
| **IL-5** | Forward | 5′-ACC TTG GCA CTG CTT TCT ACT-3′ |
|  | Reverse | 5′-CCC CTT GCA CAG TTT GAC TC-3′ |
| **IL-10** | Forward | 5′-CAT CGA TTT CTT CCC TGT GAA-3′ |
|  | Reverse | 5′-TCT TGG AGC TTA AAG GCA TTC-3′ |
| ***TSG-6*** | Forward | 5′-AGC ACG GTC TGG CAA ATA CA-3′ |
|  | Reverse | 5′-ATC CAT CCA GCA CAG AC-3′ |

Oct4, octamer-binding transcription factor 4; CXCR4, C-X-C chemokine receptor type 4; SDF-1, stromal cell-derived factor 1; COL1A1, α-1 chain of type 1 collagen; VEGF, vascular endothelial growth factor; Bcl-2, B-cell CLL/lymphoma 2; Bax, BCL2 associated X; TGF-β, transforming growth factor β, IDO-1, indoleamine 2,3-dioxygenase 1; TSG-6, tumor necrosis factor-stimulated gene 6

**Supplementary Table 2. Disease Activity Index (DAI) Scoring System**

| **Score** | **Weight loss** | **Stool consistency** | **Occult/gross rectal bleeding** |
| --- | --- | --- | --- |
| 0 | None | Normal | Normal |
| 1 | 1-5% | - | - |
| 2 | 5-10% | Loose stool | Hemoccult |
| 3 | 10-20% | - | - |
| 4 | > 20% | Diarrhea | Gross bleeding |

**Supplementary Table 3. Histological scoring system for colitis**

| **Feature score** | **Score** | **Description** |
| --- | --- | --- |
| Inflammation severity | 0 | None |
|  | 1 | Mild |
|  | 2 | Moderate |
|  | 3 | Severe |
| Inflammation extent | 0 | None |
|  | 1 | Mucosa |
|  | 2 | Submucosa |
|  | 3 | Transmural |
| Crypt damage | 0 | None |
|  | 1 | Basal 1/3 damage |
|  | 2 | Basal 2/3 damage |
|  | 3 | Crypt lost ; surface epithelium present |
|  | 4 | Crypt and surface epithelium lost |
| Percent involvement | 0 | 0% |
|  | 1 | 1–25% |
|  | 2 | 26–50% |
|  | 3 | 51–75% |
|  | 4 | 76–100% |

**Supplementary Table 4. Primer Sequences of the qRT-PCR (quantitative reverse transcriptase polymerase Chain Reaction) for mouse colon tissue.**

| **Primer** |  | **Sequence** |
| --- | --- | --- |
| *IL-1β* | Forward | 5'-GAGCCCATCCTCTGTGACTC-3' |
|  | Reverse | 5'-TCCATTGAGGTGGAGAGCTT-3' |
| *IL-6* | Forward | 5’-CCGGAGAGGAGACTTCACAG-3’ |
|  | Reverse | 5’-TCCACGATTTCCCAGAGAAC-3’ |
| *IL-17* | Forward | 5'-TCCCTCTGTGATCTGGGAAG-3' |
|  | Reverse | 5'-CTCGACCCTGAAAGTGAAGG-3' |
| *TNF-α* | Forward | 5’-ACGGCATGGATCTCAAAGAC-3’ |
|  | Reverse | 5’-AGATAGCAAATCGGCTGACG-3’ |
| *GAPDH* | Forward | 5’-TGATGACATCAAGAAGGTGGTGAAG-3’ |
|  | Reverse | 5’-TCCTTGGAGGCCATGTGGGCCAT-3’ |

IL, interleukin; TNF, tumor necrosis factor; GAPDH, glyceraldehyde-3-phosphate dehydrogenase

**Supplementary Figure 1. Size of spheroids of 3D-cultured TMSCs.** The average size of the spheroids on days 1, 2, and 3 was 182.4 ± 8.3, 169.4 ± 7.7, and 154.1 ± 4.9 μm, respectively. The size of spheroids significantly decreased after 24 h post-spheroid formation.

**Supplementary Figure 2. Expression levels of stem cell markers (Nanog, Sox2, and Oct4) in the 3D-cultured TMSCs.**


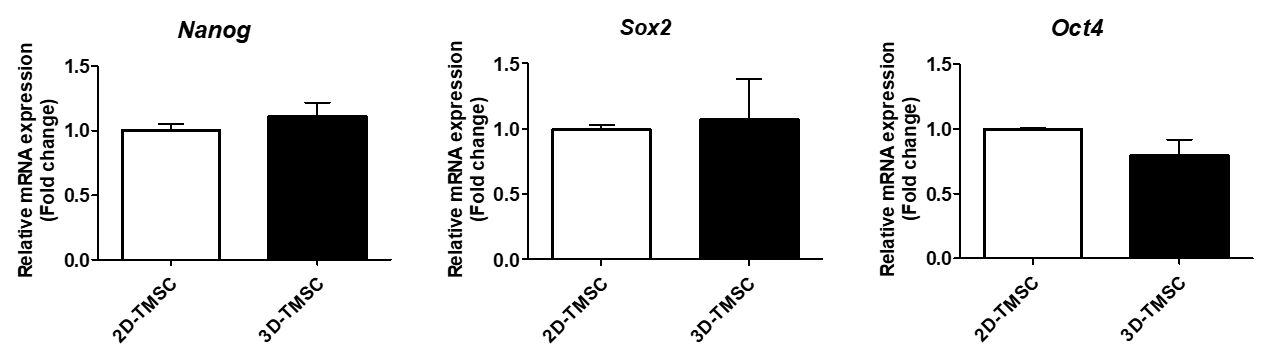

Supplement: Supplementary file 1 — Supplementary Information. [file 41598_2021_98711_MOESM1_ESM.docx]
